# Supplementary material for: Serum proteomics reveals distinct phenotypic signatures to IL-6 blockade between two immunotherapies
Source: bioRxiv. 2026 Mar 30:2026.03.27.712461. Preprint. [Version 1] doi: 10.64898/2026.03.27.712461 (PMC13060374; doi:10.64898/2026.03.27.712461)
Supplement: Supplement 1 [file NIHPP2026.03.27.712461v1-supplement-1.pdf]

bioRxiv preprint doi: https://doi.org/10.64898/2026.03.27.712461; this version posted March 30, 2026. The copyright holder for this preprint (which was not certified by peer review) is the author/funder, who has granted bioRxiv a license to display the preprint in perpetuity. It is made available under aCC-BY 4.0 International license.

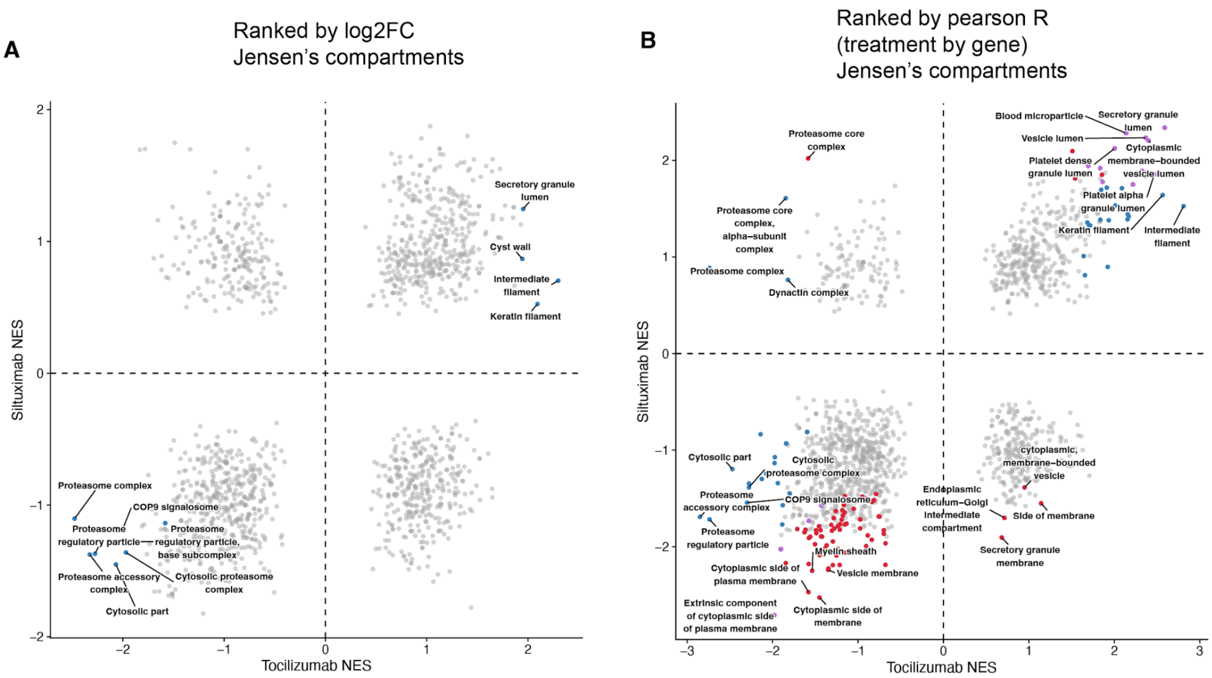

Supplemental figure 1. Fgsea analyses with Jensen's compartments at the gene set. (A) Protein groups ranked by log2FC used as input. (B) Protein groups ranked by pearson R to corresponding treatment mAb (tocilizumab or siltuximab).
